# Comparison of Oral Microbial Profile Among Patients Undergoing Clear Aligner and Fixed Orthodontic Therapies for the Treatment of Malocclusions: An Updated Review

**DOI:** 10.3390/dj13070322

**Published:** 2025-07-16

**Authors:** Emilie Ponton, Paul Emile Rossouw, Fawad Javed

**Affiliations:** Department of Orthodontics and Dentofacial Orthopedics, Eastman Institute for Oral Health, University of Rochester, Rochester, NY 14620, USA; emilie_ponton@urmc.rochester.edu (E.P.); emile_rossouw@urmc.rochester.edu (P.E.R.)

**Keywords:** clear aligner therapy, fixed orthodontics, oral microbiota, orthodontic appliances

## Abstract

**Objective**: The present review aims to compare the oral microbial profile (OMP) of patients undergoing fixed orthodontic therapy (OT) versus clear aligner therapy (CAT) for the treatment of malocclusions. **Methods**: Clinical studies were included. Case-reports/-series, letters to the editor, reviews, perspectives, and expert opinions were excluded. Indexed databases (MEDLINE/PubMed, Embase, Scopus, and Web of Science) were searched up to the end point of May 2025, without time and language barriers. The study was performed in accordance with Preferred Reporting Items for Systematic Reviews and Meta-Analyses guidelines. The risk of bias (RoB) and quality of evidence were assessed. **Results**: Three randomized clinical trials (RCTs) and seven non-RCTs were included. In all RCTs and five non-RCTs, OMP was assessed using subgingival plaque samples. Periodontopathogenic bacteria and Gram-negative anaerobic microbes were more often identified in patients undergoing fixed OT than CAT. The biofilm mass was higher in patients undergoing fixed OT than CAT. In two RCTs, periodontopathogenic bacteria were dominant among patients undergoing fixed OT than CAT. All RCTs and two non-RCTs had a high RoB. The certainty of evidence was “moderate” in 70% of the studies. **Conclusions**: Due to a high RoB, variability in study designs, and lack of power analysis, direct comparisons remain limited.

## 1. Introduction

The oral cavity hosts a complex microbial ecosystem that is essential for maintaining overall health [1]. Maintaining balance and interactions among these microbial species is essential to prevent overgrowth of opportunistic pathogenic microbes (such as bacteria and fungi) and maintain a healthy oral environment [2]. In other words, disruption of this balance (dysbiosis) may compromise oral health by allowing opportunistic pathogens to proliferate, ultimately contributing to oral diseases including carious lesions, periodontal conditions, and oral candidiasis [3,4,5].

Orthodontic tooth movement (OTM) is conventionally performed using fixed appliances, including archwires, bands and brackets, which are typically bonded to tooth surfaces for prolonged durations. The complex design of fixed orthodontic appliances often impedes effective oral hygiene practices (OHP), such as brushing and interproximal flossing, which may increase accumulation of plaque around brackets and along the gingival margin [6]. On the contrary, clear aligner therapy (CAT), which utilizes a series of removable aligners to progressively move teeth has significantly influenced clinical orthodontics and related research. Compared with fixed orthodontic therapy (OT), the removable nature of clear aligners allows patients to perform OHP, such as flossing interproximal spaces more effectively [7,8,9].

It has been reported that OTM using either fixed appliances or CAT affects the oral ecosystem and alters the microbial profile of the oral cavity [7]. Studies [10,11] have shown that patients treated with fixed OT exhibit higher levels of pathogenic microorganisms (for instance, Treponema, Porphyromonas, and *Fusobacterium* species) and increased microbial diversity compared to individuals treated with CAT. Results from a recent evidence-based review showed that the incidence of plaque deposit and cariogenic bacteria is lower in patients undergoing CAT in contrast to individuals undergoing fixed OT [12]. Despite their esthetic advantages and the convenience of removability during meals and oral hygiene practices, the close adaptation of clear aligners to the dental surfaces creates localized environments with reduced salivary flow and pH fluctuations, potentially promoting microbial shifts [7]. In this context, understanding the impact of OT (performed using fixed or removable appliances) on the oral microbial profile (OMP) is crucial for developing evidence-based strategies to mitigate treatment-associated risks of oral diseases such as those referenced above; and to tailor oral hygiene protocols among patients selected to undergo, or currently undergoing, OTM.

The present review aims to compare the OMP of patients undergoing fixed OT versus CAT for the treatment of malocclusions.

## 2. Materials and Methods

### 2.1. Ethics Statement

This study is an evidence-based review of original research articles published in peer-reviewed journals; as such, it did not require ethical approval from an institutional review board.

### 2.2. Research Question

The study addressed the following question: “is there a difference in the OMP among patients undergoing treatment for malocclusions using CAT compared to those receiving fixed OT?”

### 2.3. Eligibility Criteria

Prospective studies, cohort studies, RCTs, and descriptive cross-sectional studies that reported the OMP of patients undergoing CAT and fixed OT were included (Table 1). Case reports and case-series, letters to the editor, commentaries, reviews, in vivo/in vitro/ex vivo/in silico studies, and studies on animal models were excluded (Table 1).

### 2.4. Patients, Intervention, Control, Outcome, Study

The present study adhered to the Population (P), Intervention (I), Control (C), Outcome (O) and Study design (S) framework as follows: P: patients undergoing treatment for malocclusions; I: treatment of malocclusion with CAT; C: treatment of malocclusion with fixed OT; O: OMP; and S: clinical studies.

### 2.5. Study Selection

The following details were recorded from each included study: (1) authors; (2) study design; (3) number of participants; (4) participants’ gender; (5) participants’ age; (6) study groups; (7) treatment modality (fixed appliances or clear aligners); (8) bacteria assessed; (9) sample-size estimation/power analysis; (10) location of the microbiological sampling; (11) microbiological analysis; (12) microbiological measurement; (13) time point of the sample collection; (14) dominant bacterial groups; (15) key pathogens; and (16) microbiome shift.

### 2.6. Search Methodology

The present study was performed in accordance with the Preferred Reporting Items for Systematic Reviews and Meta-Analyses (PRISMA 2020) guidelines [13] to ensure methodological rigor and transparency. A thorough search of indexed databases (PubMed/Medline, EMBASE, Scopus, and ISI Web of Science) was performed without time and language barriers until the end May 2025. A Boolean search strategy was applied using a combination of keywords to systematically identify relevant studies. The following search terms were used across all databases: (“oral microbiota” OR “oral microbial profile” OR “oral microbiome”) AND (“fixed orthodontic therapy” OR “fixed appliances” OR “braces”) AND (“clear aligners” OR “clear aligner therapy”). Manual screening of reference lists from selected original and review articles was conducted to identify any additional relevant publications. Two independent reviewers (EP and FJ) conducted a screening of titles and abstracts for relevance. The same reviewers (EP and FJ) independently assessed the full retrieved texts for eligibility according to criteria referenced above. Any disagreements were resolved through discussion and consultation with a third investigator (PER).

### 2.7. Risk of Bias Assessment

Two authors (EP and FJ) assessed the risk of bias (RoB) of the included studies using the Cochrane Collaboration’s RoB tool [14] and the ROBINS-I [15] tool for randomized and non- randomized clinical studies, respectively. For the Cochrane Collaboration’s RoB tool [14], the RoB was considered as ‘low risk’, ‘high risk’, or ‘unclear risk’ [14]. For the non-RCTs, the RoB was considered to be ‘low’, ‘moderate’, ‘serious’, or ‘critical’, with the last type indicating either uncertainty over the potential for bias or lack of information [15]. Any disagreements in the RoB assessment were resolved as previously mentioned.

### 2.8. GRADE Analysis

The Grading of Recommendations, Assessment, Development, and Evaluations (GRADE) analysis was performed to assess the quality of evidence and the strength of recommendations [16]. The following key domains were used for GRADE analysis: (a) RoB; (b) inconsistency; (c) indirectness; (d) imprecision; and (e) publication bias. The overall quality of the evidence for each outcome was classified as “high”, “moderate”, “low”, or “very low”. The GRADE analysis was individually performed by two authors (EP and FJ). Disagreements were reconciled via discussion and consultation with a third author (PER).

## 3. Results

### 3.1. Selection of the Studies

**Figure 1:**
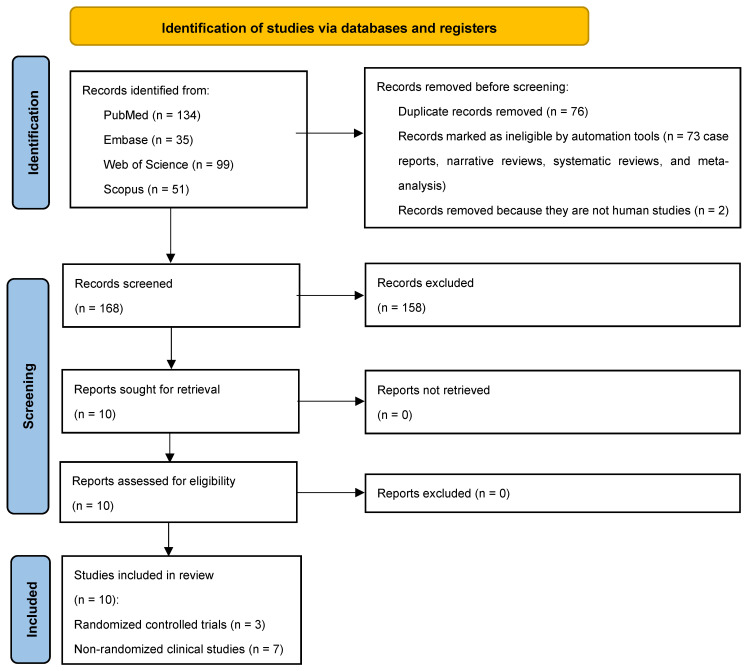
PRISMA 2020 flow diagram for scoping reviews.

Following the initial identification of 319 studies, 243 full-texts remained after duplicates (n = 76) were excluded. Seventy-three studies that were not relevant to the research question, along with two non-human studies, were further excluded. Among the 168 studies retained, 10 studies [8,11,17,18,19,20,21,22,23,24] abided by the PICO and underwent data extraction (Figure 1). Three studies were RCTs [18,19,24] and the remaining were non-randomized clinical studies [8,11,17,20,21,22,23].

### 3.2. Study Characteristics

#### 3.2.1. Randomized Controlled Trials

Three RCTs [18,19,24] were included. A total of 77, 50 and 30 individuals were assessed in studies by Levriniet al. [18], Levrini et al. [19], and Abbate, Caria et al. [24], respectively. Participant mean ages ranged from 10 to 30 years [18,19,24], and two studies [18,19] reported the gender distribution, with females comprising 68% and 30% of the patient population, respectively. In all RCTs [18,19,24], there was a presence of *Prevotella intermedia* (*P. intermedia*), *Porphyromonas gingivalis* (*P. gingivalis*, *Tannerella forsythia* (*T. forsythia*), and *Aggregatibacter actinomycetemcomitans* (*A. actinomycetemcomitans*) among patients undergoing fixed OT and CAT. In one study [19], individuals undergoing fixed OT and CAT were compared with individuals who did not receive any form of OT (10 individuals). A sample size estimation (SSE) was conducted in one RCT [18] (Table 2).

#### 3.2.2. Non-Randomized Clinical Studies

Seven non-RCTs [8,11,17,20,21,22,23] were included. All non-RCTs were prospective [8,11,17,20,21,22,23]. The total sample size ranged from 16 to 60 patients [8,11,17,20,21,22,23]. Participant mean ages ranged from 7 to 65 years [8,11,17,20,21,22,23], and six studies [8,11,17,19,20,22,23] reported gender distribution, with female participants comprising 50–100% and male participants comprising 0–48% of the sample. The number of patients who underwent CAT and fixed OT ranged from 8 to 20 and 8 to 40, respectively [8,11,17,20,21,22,23] (Table 3). In two studies [11,22], individuals undergoing fixed OT and CAT were compared with individuals who did not receive any form of OT (13 to 20 individuals). Three studies [17,20,21] assessed the presence of periodontopathic bacteria amongst which, one study [17] quantified total biofilm mass. Cenzato et al. [22] categorized bacteria on the basis of gram-staining, and Cenzato, Marcolongo et al. [23] assessed bacterial morphology (Table 3). Two studies reported conducting a SSE [21,22].

### 3.3. Microbial Sampling and Evaluation

In all RCTs [18,19,24], microbes were collected from subgingival sulci and were assessed using real-time PCR and DNA sequencing. Three [17,20,22] and two [8,11] non-RCTs, assessed microbes in samples collected from subgingival and supragingival sulci, respectively. In two non-RCTs [8,11], microbial samples were collected from supragingival and clear-aligner surfaces. Gujar et al. [21] collected microbial samples from brackets and aligner surfaces from patients undergoing fixed OT and CAT, respectively. In one study [23], the location of the microbial sample collected was not reported. Among the non-RCTs, gene sequencing and a Benzoyl-DL-Arginine-Naphthylamide test were performed in three [8,11,17] and one study [20], respectively. Gram-staining was performed in two studies [22,23] to assess the microbes (Table A1).

### 3.4. Teeth for Microbial Sampling, Periodontal Status, and Oral Hygiene Instructions

Teeth selected for microbial sample collection among patients undergoing fixed OT and CAT are presented in Table A2. In all RCTs [18,19,24], participants underwent dental prophylaxis and received oral hygiene instructions (OHI) from the provider one month before OT. All RCTs [18,19,24] reported that the periodontal statuses of fixed OT and CAT patients were comparable at baseline. Compliance towards oral hygiene maintenance (OHM) was assessed at baseline and 12 months of follow-up in two RCTs [19,24]. In one non-RCT [20], participants underwent baseline dental prophylaxis and received OHI at 1 week follow-up. Compliance towards OHM was reported in none of the non-RCTs [8,11,17,20,21,22,23]. These results are shown in Table A3.

### 3.5. Dominant Bacteria Among Patients Undergoing Fixed OT and CAT

#### 3.5.1. Randomized Controlled Trials

In two studies [18,19], periodontopathogenic bacteria including *Aggregatibacter actinomycetemcomitans* were dominant among patients undergoing fixed OT than CAT. In one RCT [24], no dominant periodontopathic anaerobes detected in patients undergoing CAT or fixed OT. In all RCTs [18,19,24], the biofilm mass was higher among patients undergoing fixed OT than CAT (Table 4).

#### 3.5.2. Non-Randomized Clinical Studies

Periodontopathogenic microbes (including red and orange complex bacteria, *Porphyromonas gingivalis*, *Prevotella* species, *Treponema denticola*, and Gram-negative bacilli and cocci) were more often isolated from samples retrieved from patients undergoing fixed OT than CAT. In all non-RCTs [8,11,17,20,21,22,23], the biofilm mass was higher among patients undergoing fixed OT than CAT [8,11,17,20,21,22,23] (Table 4).

### 3.6. RoB Assessment and GRADE Analyses

**Figure 2:**
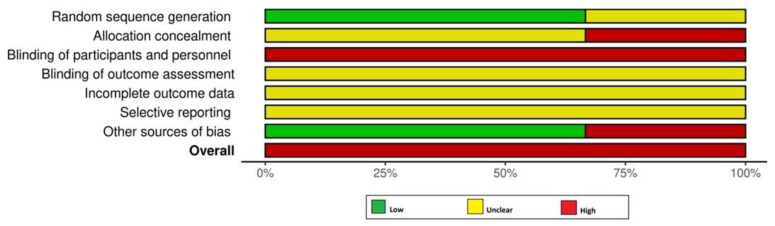
Risk of bias assessment among the randomized controlled trials.

**Figure 3:**
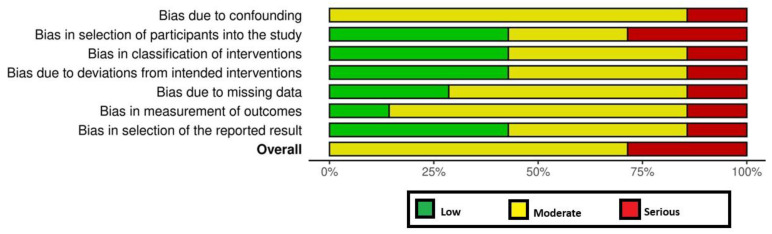
Risk of bias among the non-randomized clinical trials.

All RCTs [18,19,24] had a high RoB (Figure 2). Among the non-RCTs [8,11,17,20,21,22,23], two [21,23] and five studies [8,11,17,20,22] had a high and moderate RoB, respectively (Figure 2 and Figure 3). The certainty of evidence was very low and moderate in one [24] and two [18,19] RCTs, respectively. In the non-RCTs, the certainty of evidence was very low and low in two [21,23] and five studies [8,11,17,20,22], respectively (Table 5).

## 4. Discussion

### 4.1. Comparison with Previous Literature

A recent systematic review in French [25] with objectives similar to the present study, was identified during the literature search. This study [25] concluded that CAT is associated with a more favorable OMP and reduced biofilm accumulation compared to fixed OT. However, upon critical appraisal, several methodological limitations were noted in the systematic review by Charavet et al. [25]. Notably, this study [25] employed varying sample types, such as plaque and saliva, which introduced heterogeneity and limited the ability to perform a meta-analysis. Moreover, ten [17,18,20,21,26,27,28,29,30,31] of the eleven studies [17,18,19,20,21,26,27,28,29,30,31] studies reviewed by Charavet et al. [25] did not statistically assess baseline comparability of the CAT and fixed OT groups in terms of age and gender, thereby increasing the potential for selection bias. Similarly, in a recent quasi-experimental study, Kim et al. [32] investigated hygiene practices, oral health, and satisfaction with treatment in patients undergoing fixed OT and CAT. The results showed that while CAT may offer advantages in maintaining gingival health and reducing plaque accumulation, fixed OT patients tend to engage more in oral hygiene practices [32]. In this context, attributing the increased biofilm mass and elevated levels of periodontopathogenic microbes solely to fixed OT is possibly an overstatement. Therefore, the authors of the present study undertook a re-evaluation of the topic, incorporating these previously unaddressed parameters to enhance methodological rigor and interpretative accuracy.

### 4.2. Findings on Oral Microbiota and Methodological Limitations

A consistent finding across all studies [8,11,17,18,19,20,21,22,23,24] was that the oral biofilm mass was higher, and pathogenic bacteria were more often isolated from the oral cavities of patients in whom malocclusion was treated with fixed OT compared to those undergoing CAT. Although these outcomes appear to harmonize with the findings reported by Charavet et al. [25], several methodological inconsistencies within the included studies [8,11,17,18,19,20,21,22,23,24] may have introduced bias, thereby affecting the reliability of their results. For instance, baseline oral hygiene, particularly periodontal status, was inadequately addressed in the included studies [8,11,17,18,19,20,21,22,23,24]. All RCTs [18,19,24] reported comparable baseline periodontal status among patients undergoing CAT and fixed OT; however, such assertions lack specificity regarding whether participants were periodontally healthy, stable, or already compromised. Likewise, baseline oral hygiene/periodontal statuses remained undocumented in approximately 71% of the non-RCTs [8,11,17,20,21].

### 4.3. Role of Oral Hygiene Practices and Compliance

Kim et al. [32] reported that the frequency of professional dental prophylaxis and oral health education is higher among patients undergoing fixed OT than CAT. Moreover, oral hygiene management behaviors, particularly the duration of toothbrushing, has been reported to be higher among patients undergoing fixed OT than CAT [32]. These findings imply greater emphasis on oral hygiene reinforcement and patient compliance in fixed OT, presumably due to the enhanced plaque retention associated with fixed appliances.

It is noteworthy that information regarding adherence to OHP and routine dental checkups remained undisclosed in 80% [8,11,17,18,20,21,22,23] of the included studies [8,11,17,18,19,20,21,22,23,24]. Moreover, patients undergoing fixed OT or CAT were routinely given oral hygiene instructions in merely 40% [17,18,19,21] of the studies assessed [8,11,17,18,19,20,21,22,23,24]. The authors perceive that the absence of detailed baseline oral health/periodontal assessments in the studies systemically reviewed [8,11,17,18,19,20,21,22,23,24] undermines the potential to establish clear conclusions about the comparative effects of CAT and fixed OT on OMP.

### 4.4. Influence of Age and Population Heterogeneity

Age is a significant factor that influences OTM as well as shifts in the oral microbiome that may impact periodontal health [33,34]. According to Burcham et al. [35], the oral microbiota in adults is significantly influenced by oral hygiene practices and shows a higher presence of periodontopathic pathogens compared to children. A vigilant scrutiny of the included studies showed a considerable age variation in the RCTs [18,19,24], among which the age distribution of participants fell within the 10–30 years range. Likewise, among the non-RCTs [8,11,17,20,21,22,23], individuals aged between 7 and 65 years were included. The authors speculate that the wide variation in age range among patients undergoing fixed OT and CAT potentially biased the results regarding the OMP.

### 4.5. Quality of Evidence and Limitations

It is well established that prior SSE is essential for designing a study and eliminating the risk of errors such as Type I and Type II errors [36]. In other words, studies lacking prior power analysis may report probability values that may not necessarily reflect the true group comparisons [36]. In the present review, nearly 80% of the studies [8,11,17,19,20,23,24] lacked a prior SSE that regrettably compromises the reliability of the reported outcomes.

Furthermore, due to the methodological heterogeneity in study designs, population characteristics, sample types, and investigative parameters among the included studies [8,11,17,18,19,20,21,22,23,24], in conjunction with a high RoB and moderate certainty of evidence, meaningful quantitative synthesis of the data through meta-analysis was not feasible.

An important additional limitation is the potential for sample overlap among studies conducted by Levrini et al. [18,19,24], which share overlapping author teams, similar methodologies, and comparable outcomes. Although published in different journals and years, these studies may include data from overlapping patient populations. This possibility could compromise the independence of the data and may have impacted the robustness of the evidence synthesis and the validity of the GRADE assessment in the present review. Moreover, the included studies [8,11,17,18,19,20,21,22,23,24] employed a range of bacterial detection techniques, each capturing different dimensions of the oral microbiota. This methodological diversity limits the comparability of outcomes and complicates their clinical interpretation. It is also important to distinguish between biofilm accumulation and microbial composition, as an increase in biofilm mass does not necessarily correspond to a higher presence of pathogenic species. Another source of variability lies in the sampling sites, such as supragingival plaque, subgingival plaque, and aligner surfaces, which represent distinct microbial habitats. Although comparisons between supragingival and aligner-associated microbiota are clinically meaningful, such analyses were primarily conducted in non-RCTs [8,11].

Given these limitations, the findings of this review should be interpreted with caution. Future research should prioritize well-designed, power-adjusted studies with standardized methodologies, clearly defined populations, and uniform outcome measures to evaluate the precise influence of CAT and fixed OT on OMP, thereby enabling potential meta-analyses.

### 4.6. Clinical Implications and Recommendations

The authors suggest that it is essential that clinicians actively educate patients (including those currently undergoing and those scheduled to undergo fixed OT or CAT) on the importance of adhering to daily oral hygiene practices, attending routine dental examinations, and receiving regular professional prophylaxis as recommended by their oral healthcare providers. Such preventive measures are vital not only for minimizing biofilm accumulation on teeth and orthodontic appliances but also for maintaining a balanced oral microbial ecosystem, thereby reducing the risk of microbial dysbiosis and its associated complications.

## 5. Conclusions

Due to methodological heterogeneity, a high RoB, and only moderate certainty of evidence across the included studies, it is challenging to draw definitive conclusions regarding the impact of fixed OT and CAT on OMP. This underscores the need for further well-designed and power-adjusted studies.

## Figures and Tables

**Table 1:** Eligibility criteria.

Inclusion Criteria	Exclusion Criteria
Prospective studies, cohort studies, RCTs and descriptive cross-sectional studies that reported the OMP of patients undergoing CAT and fixed OT.	Case reports and case-series, letters to the editor, commentaries, reviews, in vivo/in vitro/ex vivo/in silico studies and studies on animal models.

RCT, randomized controlled trials; FOT, fixed orthodontic therapy; CAT, clear aligner therapy.

**Table 2:** General characteristics of randomized controlled trials.

Authors et al.	Patients (n)	Gender (M:F) (n)	Mean Age (Range)	Groups (n)	Bacteria Assessed
Levrini et al. [18]	77	Group 1: (5:27) Group 2: (18:17) Group 3: (2:8)	24.3 ± NR years (16–30)	Group 1: CAT (32) Group 2: FOT (35) Group 3: Control (10)	- P. intermedia - A. actinomycetemcomitans - P. gingivalis - T. forsythia
Levrini et al. [19]	30	Group 1: (3:7) Group 2: (3:7) Group 3: (3:7)	Group 1: 24.6 ± 6.4 years (NR) Group 2: 25.7 ± 3.4 years (NR) Group 3: 25.0 ± 3.4 years (NR)	Group 1: CAT (10) Group 2: FOT (10) Group 3: Control (10)	- P. intermedia - A. actinomycetemcomitans - P. gingivalis - T. forsythia
Abbate et al. [24]	50	NR	NR (10–18)	Group 1: CAT (22) Group 2: FOT (25)	- P. intermedia - A. actinomycetemcomitans - P. gingivalis - T. forsythia

FOT, fixed orthodontic therapy; CAT, clear aligner therapy; SD, standard deviation; M, male; F, female; NR, not reported; F., *Fusobacterium*; P., *Prevotella*; P., *Porphyromonas*; T., *Tannerella*; T., *Treponema*; A., *Aggregatibacter*; C., *Campylobacter*.

**Table 3:** General characteristics of included non-randomized clinical studies.

Study Author	Patients (n)	Gender (M:F) (n)	Mean Age (Range)	Groups (n)	Bacteria Assessed
Shokeen et al. [8]	24	Group 1: (4:8) Group 2: (4:8)	Group 1: 29 ± 12 years (NR) Group 2: 22 ± 13 years (NR)	Group 1: CAT (12) Group 2: FOT (12)	- Streptococcus - Actinomyces - Corynebacterium - Capnocytophaga - Leptotrichia - Neisseria - Rothia - Fusobacterium - Prevotella - Veillonella - Lautropia - Haemophilus - Porphyromonas - Pseudopropionibacterium - Selenomonas - Granulicatella - Saccharibacteria - Cardiobacterium - Campylobacter - Kingella - Aggregatibacter - Abiotrophia - Gemalla
Zheng et al. [11]	48	(0:48)	Group 1: 27.9 ± 5.2 years (NR) Group 2: 26.2 ± 6.0 years (NR) Group 3: 26.0 ± 6.6 years (NR)	Group 1: CAT (18) Group 2: FOT (17) Group 3: Control (13)	- Lautropia - Rothia - Prevotella - Fusobacterium - Leptotrichia - Neisseria - Capnocytophaga - Veillonella - Haemophilus - Streptococcus - Actinobacteria - Bacteroidia - Burkholderiacese - Flavobacteriaceae - Prevotellaceae - Saccharimonadaceae
Lombardo et al. [17]	27	Group 1: (5:9) Group 2: (5:8)	Group 1: 21 ± 0.25 years (NR) Group 2: 14 ± 0.75 years (NR)	Group 1: CAT (14) Group 2: FOT (13)	- A. actinomycetemcomitans - P. gingivalis - F. nucleatum - C. rectus - T. denticola - T. forsythia
Karkhanechi et al. [20]	42	Group 1: (8:12) Group 2: (6:16)	Group 1: 28.0 ± 6.86 years (18–44) Group 2: 34.0 ± 7.18 years (18–44)	Group 1: CAT (20) Group 2: FOT (22)	- T. denticola - P. gingivalis - T. forsythia - Gram-negative anaerobic bacteria
Gujar et al. [21]	60	NR	NR (11–29)	Group 1: CAT (20) Group 2: FOT (40)	- C. rectus - F. nucleatum - F. periodontium - P. intermedia - P. melaninogenica - P. gingivalis - T. forsythia - T. denticola
Cenzato et al. [22]	60	Group 1: (11:8) Group 2: (9:11) Group 3: (9:11)	Group 1: 20 years (12–65) Group 2: 18 years (12–65) Group 3: 43 years (12–65)	Group 1: CAT (20) Group 2: FOT (20) Group 3: Control (20)	- Cocci - Bacilli - Spirochetes - Filamentous bacteria - Fusiform bacteria
Cenzato et al. [23]	16	(7:9)	Group 1: 14.5 years (7–35) Group 2: 15.7 years (7–35)	Group 1: CAT (8) Group 2: FOT (8)	- Gram-positive cocci - Gram-negative cocci - Gram-positive bacilli - Gram-negative bacilli

Abbreviations: FOT, fixed orthodontic therapy; CAT, clear aligner therapy; SD, standard deviation; M, male; F, female; NR, not reported; F., *Fusobacterium*; P., *Prevotella*; P., *Porphyromonas*; T., *Tannerella*; T., *Treponema*; A., *Aggregatibacter*; C., *Campylobacter*.

**Table 4:** Main study outcomes regarding the oral microbial profile.

Authors et al.	Dominant Bacterial Groups in FOT vs. CAT	Key Pathogens in FOT vs. CAT	Microbiome Shift in FOT vs. CAT	Conclusion (FOT vs. CAT)
Randomized Controlled Trials				
Levrini et al. [18]	More periodontopathogens in FOT.	More *A. actinomycetemcomitans* in FOT (one patient).	Shift to more pathogenic microbiome in FOT.	Higher biofilm mass in FOT.
Levrini et al. [19]	More periodontopathogens in FOT.	More *A. actinomycetemcomitans* in FOT (one patient).	Shift to more pathogenic microbiome in FOT.	Higher biofilm mass in FOT.
Abbate et al. [24]	No dominant periodontopathogenic anaerobes detected in both groups.	No dominant periodontopathic anaerobes detected in both groups.	Participant tested negative for periodontopathogens at all time points.	Higher biofilm mass in FOT.
Non-Randomized Clinical Studies				
Shokeen et al. [8]	More anaerobes, Gram-negative and orange complex bacteria in FOT.	More *Leptotrichia*, *Prevotella*, *Veillonella*, *Selenomonas*, *Campylobacter*, and *Saccharibacteria (TM7)* in FOT.	Shift to beta diversity microbiome in FOT.	Higher biofilm mass in FOT.
Zheng et al. [11]	More anaerobes and Gram-negative bacteria in FOT.	More *Prevotella*, *Veillonella*, and *Capnocytophaga* in FOT.	Shift to more pathogenic microbiome in FOT.	Higher biofilm mass in FOT.
Lombardo et al. [17]	More orange complex bacteria in FOT.	More *C. rectus*, *F. nucleatum* in FOT.	Shift to more pathogenic microbiome in FOT.	Higher biofilm mass FOT.
Karkhanechi et al. [20]	More periodontopathogens in FOT.	More *P.gingivalis*, *T. forsythia*, and *T. denticola* in FOT.	Shift to more pathogenic microbiome in FOT.	Higher biofilm mass in FOT.
Gujar et al. [21]	More red and orange complex bacteria in FOT.	More *T. denticola*, *F. nucleatum*, *T. forsythia*, and *P. gingivalis* in FOT.	Shift to more pathogenic microbiome in FOT.	Higher biofilm mass in FOT.
Cenzato et al. [22]	More bacilli and cocci in FOT.	More Gram-negative and anaerobic morphotypes in FOT.	Shift to more pathogenic microbiome in FOT.	Higher biofilm mass in FOT.
Cenzato et al. [23]	More Gram-negative bacilli and cocci and less Gram-positive cocci in FOT.	More Gram-negative cocci and bacilli in FOT.	Shift to more pathogenic microbiome in FOT.	Higher biofilm mass in FOT.

FOT, fixed orthodontic treatment; CAT, clear aligner therapy; F., *Fusobacterium*; P., *Prevotella*; P., *Porphyromonas*; T., *Tannerella*; T., *Treponema*; A., *Aggregatibacter*; C., *Campylobacter*.

**Table 5:** GRADE analysis.

Authors et al.	Microbial Analysis	Study Design	Risk of Bias	Inconsistency	Indirectness	Imprecision	Publication Bias	Certainty of Evidence
**Randomized Controlled Trials**								
Levrini et al. [18]	Real-time PCR	RCT	Moderate	Low	Low	Moderate	Moderate	●●●○ Moderate
Levrini et al. [19]	Real-time PCR	RCT	Moderate	Low	Low	Moderate	Moderate	●●●○ Moderate
Abbate et al. [24]	Real-time PCR	RCT	High	Moderate	Low	High	High	●○○○ Very low
**Non-randomized clinical trials**								
Shokeen et al. [8]	16S rRNA sequencing	Non-RCT	Moderate	Moderate	Moderate	Moderate	Moderate	●●○○ Low
Zheng et al. [11]	16 S rRNA sequencing	Non-RCT	Moderate	Moderate	Low	Moderate	Moderate	●●○○ Low
Lombardo et al. [17]	Real-time PCR	Non-RCT	Moderate	Moderate	Moderate	Moderate	Moderate	●●○○ Low
Karkhanechi et al. [20]	BANA test	Non-RCT	Low	Moderate	Low	Low	High	●●○○ Low
Gujar et al. [21]	CDDH	Non-RCT	Moderate	High	Moderate	Moderate	High	●○○○ Very low
Cenzato et al. [22]	Gram staining	Non-RCT	Moderate	Moderate	Moderate	Moderate	Moderate	●●○○ Low
Cenzato et al. [23]	Gram staining	Non-RCT	Moderate	High	Low	Moderate	Moderate	●○○○ Very low

Abbreviations: CDDH, checkerboard DNA–DNA hybridization; PCR, polymerase chain reaction; RCT, randomized clinical trial; RNA, ribonucleic acid.

## Data Availability

Data is available on reasonable request.

## Abbreviations

The following abbreviations are used in this manuscript:

CAT	Clear aligner therapy
GRADE	Grading of Recommendations, Assessment, Development, and Evaluations
OHI	Oral hygiene instructions
OHP	Oral hygiene practices
OMP	Oral microbial profile
OT	Orthodontic therapy
OTM	Orthodontic tooth movement
RCTs	Randomized controlled trials
RoB	Risk of bias
ROBINS	Risk of bias for non-randomized studies

## Appendix A

**Table A1:** Study characteristics related to the assessment of bacteria.

Authors et al.	Location of Sample Collection	Microbiological Analysis	Microbiological Measurement	Time Point Of The Sample Collection
Randomized Controlled Trials				
Abbate et al. [24]	Subgingival sulcus	Real-time PCR; DNA sequencing	Periodontopathic bacteria	T0: Tx start T1: 3 months T2: 6 months T3: 12 months
Levrini et al. [18]	Subgingival sulcus	Real-time PCR; DNA sequencing	Total biofilm mass and presence of periodontopathic bacteria	T0: Tx start T1: 1 month T2: 3 months
Levrini et al. [19]	Subgingival sulcus	Real-time PCR; DNA sequencing	Total biofilm mass and periodontopathic bacteria	T0: Tx start T1: 1 month T2: 3 months
Non-randomized Clinical Studies				
Shokeen et al. [8]	Supragingival and CA	16 S rRNA gene sequencing	Bacterial composition and diversity	T0: Tx start T1: 1 month T2: 3 months T3: 6 months T4: 12 months
Zheng et al. [11]	Supragingival and CA	16 S rRNA gene sequencing	Bacterial composition, diversity, and functional characteristics	NR
Lombardo et al. [17]	Subgingival sulcus	Real-time PCR; DNA sequencing	Total biofilm mass and periodontopathic bacteria	T0: before Tx start T1: 1 month T3: 3 months T6: 6 months
Karkhanechi et al. [20]	Subgingival sulcus	BANA test	Presence of periodontopathic bacteria	T0: before Tx start T1: 6 weeks T2: 6 months T3: 12 months
Gujar et al. [21]	Brackets and CA	CDDH	Periodontopathic bacteria	T: 30 days
Cenzato et al. [22]	Subgingival sulcus	Gram staining; microscopic examination	Bacterial morphology	T: 1 year
Cenzato et al. [23]	NR	Gram staining; microscopic examination	Bacterial composition (gram+, gram-;) and morphology	NR

Abbreviations: FOT, fixed orthodontic therapy; CAT, clear aligner therapy; CA, clear aligner; CDDH, checkerboard DNA–DNA hybridization; NR, not reported.

**Table A2:** Teeth used for sample collection.

Authors et al.	FOT (Teeth) *	CAT (Teeth)
Randomized Controlled Trials		
Levrini et al. [18]	Maxillary right first molar and left central incisor (16, 21)	Maxillary right first molar and left central incisor (16, 21)
Levrini et al. [19]	Maxillary right first molar and left central incisor (16, 21)	Maxillary right first molar and left central incisor (16, 21)
Abbate et al. [24]	Maxillary right first molar and left central incisor (16, 21)	Maxillary right first molar and left central incisor (16, 21)
Non-randomized Clinical Studies		
Shokeen, et al. [8]	NR	NR
Zheng et al. [11]	Maxillary left second molar to mandibular right second molar (27–47)	Maxillary left second molar to mandibular right second molar (27–47)
Lombardo et al. [17]	Maxillary right first molar and central incisor (11, 16)	Maxillary right first molar and central incisor (11,16)
Karkhanechi et al. [20]	Maxillary first molars, lateral incisors, central incisors and left canine (16, 12, 11, 21, 22, 23, 26)	Maxillary first molars, lateral incisors, central incisors and left canine (16, 12, 11, 21, 22, 23, 26)
Gujar et al. [21]	NR **	NR **
Cenzato et al. [22]	Mandibular left central incisor (31)	Mandibular left central incisor (31)
Cenzato et al. [23]	Mandibular right first molar (46)	Mandibular right first molar (46)

Abbreviations: FOT, fixed orthodontic therapy; CAT, clear aligner therapy; NR, not reported. * FDI World Dental Federation notation was used. ** Gujar et al. only mentioned the maxillary arch without specifying the specific tooth used.

**Table A3:** Baseline oral hygiene status and related instructions.

Authors et al.	Initial Professional Prophylaxis	Oral Hygiene Instructions	Oral Hygiene Compliance	Baseline Periodontal Measurements
Randomized Controlled Trials				
Levrini et al. [18]	Yes (1 month before T0)	Yes (each appointment)	NR	PI, PD, and BOP similar between FOT, CAT, and control group (measurement reported only for BOP)
Levrini et al. [19]	Yes (1 month before T0)	Yes (each appointment)	Yes (T0, 1 month and 3 months)	PI, PD, and BOP similar between FOT, CAT, and control group (measurements reported)
Abbate et al. [24]	Yes (1 month before T0)	Yes (1 month before T0)	Yes (T0 and 12 months)	FMBS, PI, and PD similar between FOT and CAT FMPS and BOP different between FOT and CAT (measurements reported)
Non-Randomized Clinical Studies				
Shokeen et al. [8]	NR	NR	NR	PI and GI similar between FOT and CAT (measurements reported in FOT)
Zheng et al. [11]	NR	NR	NR	NR
Lombardo et al. [17]	NR	Yes (each appointment)	NR	PPD <4 mm and BOP <10% for all participants (no measurements reported)
Karkhanechi et al. [20]	Yes (1 week before T0)	Yes (T0)	NR	PI, GI, BOP and PPD similar between FOT and CAT (no measurement reported)
Gujar et al. [21]	NR	Yes (each appointment)	NR	GI < 1 and PD ≤ 3 mm for all participants (no measurement reported)
Cenzato et al. [22]	NR	Yes (NR)	NR	FMPS and PI significantly higher in control than CAT PSR significantly higher in control and FOT than CAT mSB and FMBS similar between FOT, CAT and control group (measurements reported)
Cenzato et al. [23]	NR	Yes (NR)	NR	BI significantly higher in FOT than CAT (measurements reported)

Abbreviations: FOT, fixed orthodontic therapy; CAT, clear aligner therapy; NR, not reported; PI, plaque index; BI, bleeding index; GI, gingival index; PD, probing depth; BOP, bleeding on probing; PPD, probing pocket depth; FMPS, full-mouth plaque score; FMBS, full-mouth bleeding score; mSBI, modified sulcus bleeding index; PSR, periodontal screening and recording.
